# The prevalence and its associated factors of psychological stress among middle school students in China: pooled evidence from a systematic scoping review

**DOI:** 10.3389/fpubh.2024.1358210

**Published:** 2024-04-17

**Authors:** Li Hao, Arimi Fitri Mat Ludin, Mahadir Ahmad, Xie Meng, He Zhong Lei

**Affiliations:** ^1^Center for Healthy Ageing and Wellness (HCARE), Faculty of Health Sciences, Universiti Kebangsaan Malaysia, Kuala Lumpur, Malaysia; ^2^Nan Hang Secondary School, Nanjing, China; ^3^Center for Community Health Studies (ReaCH), Faculty of Health Sciences, Universiti Kebangsaan Malaysia, Kuala Lumpur, Malaysia; ^4^Nan Jing Qin Huai Teachers Development Centre, Nanjing, China

**Keywords:** psychological stress, middle school, students, China, scoping review

## Abstract

Psychological issues are of significant concern in present-day society, as poor mental well-being results in depression and suicidal behavior. Understanding the current situation of psychological stress among secondary school students will help policy makers to formulate targeted measures to help them cope with stress, and at the same time evaluate the effectiveness of the existing policies to address the shortcomings and enhance the diversification of interventions. The main purpose of this review was to map the existing evidence on the prevalence and levels of psychological stress among adolescents in China, and to identify the associated risk factors. This review strictly adhered to the Preferred Reporting Items for Systematic Reviews and Meta-Analyses (PRISMA). A comprehensive search was performed spanning Web of Science, PubMed, and Scopus databases. Studies involving only humans and full text in English were selected. Selection was limited to samples from mainland China, Hong Kong, Macau, and Taiwan. Variables were extracted, exploring the factors that affected the mental wellness of Chinese middle school students. A final 15 articles and 1 report were included. The findings revealed psychological stress is prevalent among Chinese middle school population, with the degree of prevalence from low to severe stress levels. Three dimensions of psychological stress affecting Chinese secondary school students: school, family and lifestyle. Within the school, the factors included academic stress, peer relationships etc. Family-related factors were comprised of parent-child relationship, parents’ mental health status etc. Lastly, lifestyle-related factors included poor diet, sedentary and inactivity etc. Our findings suggest that policy makers should reduce the excessive emphasis on examination results and focus on the all-round development of students, and that schools should organize a variety of extra-curricular activities to reduce students’ stress. Parents should create a harmonious family atmosphere to minimize conflicts and maintain close communication with teachers.

**Systematic Review Registration:**

OSF; https://doi.org/10.17605/OSF.IO/HEFCP.

## 1 Introduction

Psychological stress is a widely recognized term that represents the processes believed to impact the emergence and continuation of diverse mental and physical conditions. Despite considerable interest in psychological stress and its implications for health and overall welfare, how to best define the term remains a debate. Stress is characterized by emotional strain and pressure, representing a form of psychological discomfort. Moderate levels of stress (eustress) can be advantageous, enhancing individual performance, motivation, and responsiveness to the environment. Conversely, excessive stress (distress) may elevate the risk of conditions like strokes, heart attacks, ulcers, and mental disorders such as depression. In this review, we focus on negative psychological stress. Psychological stress is considered as a precursor to anxiety and depression (1).

Psychological stress has the potential to adversely predict the academic achievement of adolescents (2). In various Western contexts, several studies have shown a correlation between high levels of academic stress and increased psychological stress levels (3). Prolonged exposure to psychological and environmental burden is widely recognized as a trigger for physical symptoms, such as heightened pulse, raised blood pressure, and metabolic disruption. If without proper treatment, these symptoms expedite the progression of cardiovascular disease, diabetes, and various health issues associated with aging (4). Adolescence is considered an important period for psychological development, during which adolescents experience various psychological stresses (5, 6). The middle school phase represents a critical time for the shaping and maturation of one’s personality. Emotional experiences, life events and educational experiences during this period will greatly influence their personality development. Excessive psychological stress has been demonstrated to correlate with numerous unfavorable outcomes. Previous studies indicate a positive relationship between accelerating stress levels and declining health (7). Moreover, adolescents experiencing psychological stress showed an adverse correlation with their sleep quality (8), and excessive psychological stress could potentially elevate the risk of experiencing depression (9, 10). Some studies examining psychological stress discovered that it depleted self-control resources, posed a risk for heightened anxiety, and significantly and adversely impacted life satisfaction (11, 12). Presently, psychological stress is prevalent globally and ranks as the most widespread mental health issue. According to the WHO data (13), approximately 14% of individuals aged 10–19 worldwide encounter a mental disorder, yet it remains largely unrecognized and untreated. At the same time, it is more severe among older adolescents than in their younger counterparts. Chinese adolescents encounter significant psychological stress. In contrast to Western nations like the USA and Australia, students in China dedicate more time to studying and completing homework (14). An investigation involving 1,576 secondary students in China revealed that a notable percentage of the student population exhibited symptoms of psychological stress that significantly impacted fundamental aspects of their daily functioning, such as sleep, and affected their overall quality of life (15). Studies suggest a marked increase in the prevalence of psychological stress symptoms among Chinese middle school students with advancing age, escalating from 24.5% during the grade 7–40.1% by the grade 12 (16).

Several literature reviews have attempted to consolidate the psychosocial factors that influence stress in adolescents. One study noted that psychological stress in middle school students can lead to unsafe behaviors, enhancing the overall safety climate on campus can effectively relieve students’ psychological stress, thereby reducing the likelihood of unsafe behaviors occurring (17). Academic stress has been identified as a factor associated with psychological problems among adolescents (18) and younger adults’ musculoskeletal pain (19). Meanwhile, negative life events have the potential to initiate psychological stress, thereby affecting the mental health of adolescents (20). Notably, adolescents’ anxiety and depression showed a positive correlation with mobile phone addiction (21), as well as university students’ increased risk of stress with more hours spent on internet gaming (22). Students under significant academic pressure face an increased likelihood of developing psychological stress (23). Additionally, a number of studies indicate a connection between physical activity and psychological stress, self-esteem, cognitive functioning, and its role in preventing and managing non-communicable diseases like obesity, cardiovascular disease, cancer, and diabetes (24) as well as addressing poor sleep quality (25, 26). Some studies have indicated that inadequate parent-child relationships (27), and poor-quality communication between parents and children were associated with increased depressive symptoms in adolescents from China (28). Increased parental warmth typically diminished the connection between parent pressure and adolescent’s psychopathology symptoms (29). Consequently, this review aimed to scope the information on psychological stress among middle school students in China. This will help to identify the sources of psychological stress among Chinese middle school students and develop strategies to reduce psychological stress among Chinese middle school students.

## 2 Methodology

This scoping review was prepared based on the Arksey and O’Malley (30) framework and conducted by Preferred Reporting Items for Systematic Reviews and Meta-Analysis extension for Scoping Review (PRISMA) guidelines (31). The protocol for this review has been registered on the Open Science Framework platform (32).

### 2.1 Identify the research question

The main purpose of this study was to address the following questions:

- Is there existing evidence of widespread psychological stress among Chinese middle school students and what are their psychological stress levels?
- What are the commonly used tools in assessing psychological stress levels among Chinese middle school students?
- What are the risk factors contributing to psychological stress among adolescents in China?

### 2.2 Identify relevant studies

This study involved searching through the following databases: PubMed, Web of Science, and Scopus. Cross-referencing was utilized to identify studies by employing varied combinations of search terms. We adopted the P (Population), C (Concept), and C (Context) to identify keywords and their synonyms. The following search terms and combinations formed the search strategy: (“middle school” OR “secondary school” OR “junior high school” OR student) AND (“psychological stress” OR “life stress” OR “mental stress” OR pressure OR “student life stress”) AND China. Table 1 displays the specific search terms employed during the database search process.

**Table 1:** The search terms used for the database search.

Population	Concept	Context
Middle school	Psychological stress	China
Secondary school	Life stress	
Junior high school	Mental stress	
Student	Pressure	
	Student life stress	
	Academic stress	

### 2.3 Study selection

#### 2.3.1 Inclusion criteria

The identified articles were selected based on inclusion criteria. Only articles published between Jan 2005 to Jan 2023 and reported adolescent psychological stress were included. The type of study should be cross-sectional. The participants in the reported study must be between 12 and 18 years of age. The studies were conducted in China, Hong Kong, Macao, and Taiwan. The study must report the psychological stress and be published in English.

#### 2.3.2 Exclusion criteria

We excluded the study if it was conducted after a natural disaster, such as an earthquake or COVID-19 pandemic. We also excluded any reports, conference papers, commentaries, editorials, meta-analyses, systematic reviews, clinical studies, and studies that were still in the formative phase.

All selected articles were uploaded into Rayyan (33) and duplicate articles were removed. Two researchers screened the title and abstract independently. The articles were only included when both researchers agreed, and a third independent researcher was referred to resolve any conflict of opinions between the initial two researchers.

### 2.4 Charting the data

A data table was generated using Microsoft Excel, which contained the details about the authors, publication year, sample characteristics, study design, prevalence, risk factors, outcomes, limitations, and title.

### 2.5 Collating, summarizing, and reporting results

We collated and mapped publications to include results relevant to psychological stress among middle school students in China. We developed a framework to group the factors that caused psychological stress among middle school students in China into several main categories.

## 3 Results

**Figure 1:**
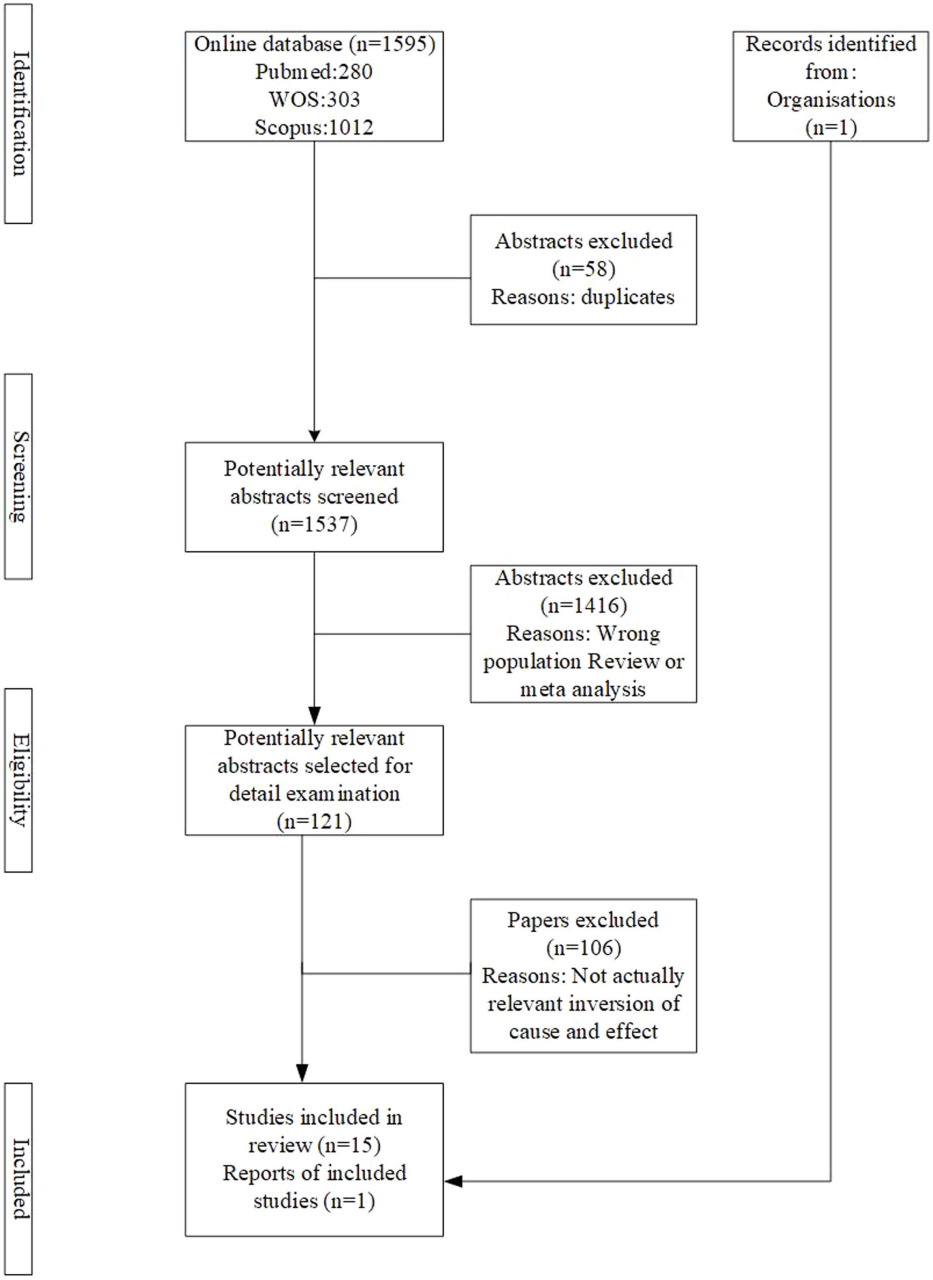
The PRISMA flow diagram for the articles selection process.

A total of 1,595 articles were retracted from the three selected databases and one report from the website. After the removal of duplicates, 1,537 articles were screened by the title and abstract. Subsequently, 121 articles were selected for full-text reading. Finally, there were 16 studies met the criteria for the research (Figure 1).

Of the 16 studies, five were published in 2020, five were published in 2021, and the remaining six were distributed in 2012–2022. Fifteen studies were conducted in mainland China, covering most of mainland China, including urban and rural, while the other study was conducted in Taiwan, China. All studies had relatively large sample sizes. The minimum sample size was 350 and the maximum sample size was 14,356. The sample consisted primarily of students in grades 7 through 12, ranging in age from 12 to 18. Among the 16 studies included in this review, five used the mediation model analysis.

### 3.1 Prevalence and stress level

Four studies (20, 23, 34, 35) reported on the prevalence of psychological stress among Chinese adolescents, with the prevalence rates being 26.6%; 21.37%; 24.78%; and 12.9–23.2%, respectively. One report (36) from the Chinese government stated that the prevalence of psychological stress among secondary school students ranged from 12.9 to 26.6%. The rest of the articles did not report it. Three studies (18, 20, 37) showed that the students’ psychological stress levels were severe, five studies (17, 21, 34, 38, 39) reported moderate stress levels, and five (23, 40–43) were low-stress level. However, three studies did not report the stress level.

### 3.2 Assessment tools

Five studies used the DASS-21 questionnaire as a stress assessment tool, two studies used the Strengths and Difficulties Questionnaire (SDQ) as a stress assessment tool. Mental Health scale, Mental Health Test (MHT), Revised Children’s Manifest Anxiety Scale (RCMAS), Multidimensional Sub-health Questionnaire of Adolescents (MSQA), Social Anxiety Scale for Children (SASC), Kessler Psychological Distress Scale (K6), and 5-item Brief Symptom Rating Scale (BSRS-5) were used once among the 15 studies. One study did not show an assessment tool.

### 3.3 Risk factors for psychological stress

In total, three categories addressed the factors related to psychological stress that affected Chinese middle school students: school category (*n* = 9), family category (*n* = 10), and lifestyle (*n* = 4). School categories included academic stress (20, 23, 43), peer relationship (20, 38), teacher-student relationship (41), campus safety atmosphere (17), teacher’s mental health (34), and school mental health education (23). Family categories included parent-child relationship (18, 20, 38, 43, 44), parental relationship (44), family financial situation (40, 44), parental mental health status (34), and student’s childhood experience (20). Lifestyle factors included dietary habits, exercise habits, and sedentary behavior (35), internet addiction, and mobile phone addiction (21, 37, 42). The main school-wide influencing factors were academic stress, teacher-student relationship, peer relationship, campus safety atmosphere, teachers’ mental health, and psychological education situation in schools. Family factors mainly included the parent-child relationship, the parent’s marital status, the family’s financial situation, parental mental health, and the student’s childhood experience. An unhealthy lifestyle was another factor that affected students’ psychological pressure, such as mobile phone and Internet addiction that led to students’ psychological stress.

### 3.4 School-related factors

Influenced by Asian culture, particularly Confucianism, parents in Asian countries often prioritize their children’s education, academic success, and adherence to cultural values. Chinese adolescents pay a lot of attention to education, dedicating their time and energy to achieving exceptional academic performance to enhance their prospects for development. Therefore, Chinese students spend most of their time at school. According to the two national large-scale surveys conducted by the China Youth Research Center between 2010 and 2015 on the subject of “China’s Children’s Development,” the average school time for primary school students increased from 6.7 h in 2010 to 8.1 h; the average school time for middle school students increased from 7.7 h to 11 h, with most of the time at school is spent for studying and doing homework. Chinese students, therefore, have to deal with increasing academic pressure throughout their adolescence (18, 23, 43). In our study, academic stress was the most widespread contributor to students’ psychological stress.

In the context of schools, students spend more time with teachers, playing a significant part in the process of students’ growth. Teachers can offer adolescents varying forms of support, including emotional, academic, and competence-based assistance, all of which help to reduce the psychological pressure on the students. That is to say, students who receive greater support from teachers commonly demonstrate increased self-awareness and maintain a positive self-assessment. Hence, they could undergo a decrease in negative emotional experiences, specifically experiencing a decrease in levels of depression (34, 41). Thus, a good teacher-student relationship and a good psychological state of teachers are very important to the mental health of students. Compared with teachers, students spend more time studying and living with their peers than teachers. Students who have developed more caring and healthy relationships with their peers may be more likely to have higher levels of mindfulness, and this higher capacity for reflection to remain mindful of what is happening can be a psychological resource for them (38). All these resources, in turn, could assist students in adopting more open and adaptable perspectives when managing stress. In other words, a harmonious peer relationship helps students perceive mindfulness and reduce psychological stress. Another interesting finding in our research is that the safety environment on campus is perceived as supportive in alleviating students’ mental stress (17). This issue concerns school violence, causing both physical harm and a range of psychological health issues. Among various educational stages where school violence and bullying incidents occur, middle school students notably face a significant prevalence of bullying within their school environment.

### 3.5 Family-related factors

The family is a child’s initial educational environment, with parents playing the role of the child’s primary educators. This highlights the importance of family in the overall development of students. On the one hand, the close bond and positive communication between parents and children can cultivate feelings of love and care in children, actively promoting their development and overall sense of well-being. On the other hand, conflict between parents and children has the potential to weaken children’s sense of closeness and belonging to their parents and within the family. As a result, this can harm their mental health and lead to increased psychological stress. In our review, we found that not only the parent-child relationship impacted students’ psychological well-being, but also the relationship between parents impacted student’s mental health. Consequently, a high-quality marital relationship between parents and a positive parent-child relationship would help improve students’ mental health. The contrary will increase students’ psychological stress. However, research findings indicate that disharmonious parent-child relationships are prevalent in China. More than 80% of adolescents reported negative experiences in their relationship with their parents (18, 34, 43, 44). Another finding of the review was that family income significantly influenced children’s mental health (40), and high family financial status predicts a positive mental health outcome. Childhood experiences (39) were also a factor that affected students’ psychological stress.

### 3.6 Lifestyle-related factors

Our present review found that unhealthy lifestyles were associated with psychological stress in students. There was a positive association found between an unhealthy lifestyle and the manifestation of psychological problems (35), and the lack of exercise and sedentary behavior can threaten students’ well-being. We also found that insufficient sleep and excessive video viewing were associated with an increased risk of developing psychological symptoms. Research confirms that psychological symptoms related to depression and anxiety are becoming more prevalent among obese children and adolescents. In addition, skipping breakfast and consuming sugary beverages above the normal limit have also been identified as factors contributing to an increased risk of obesity. Therefore, adolescents with poor eating habits are more likely to develop psychological problems.

With the popularity of mobile phones, life has become more convenient, and students’ studies and life have been closely connected with the mobile phone. However, several problems have arisen as a result, such as an overuse of mobile phones and mobile phone addiction. Increasing evidence indicates a heightened risk of mobile phone addiction among adolescents. Furthermore, some findings suggest a potential causal link between mobile phone addiction and mental health issues. The outcomes of a three-year longitudinal study highlighted that mobile phone addiction during 8th grade served as a significant predictor of depressive symptoms by 10th grade. Furthermore, a substantial and positive correlation was identified between mobile phone addiction and psychological stress. In a study conducted in mainland China and Taiwan, it was observed that cyber victimization played a crucial role in contributing to psychological stress. With the popularity of information technology, the internet has become an important link for students to learn and communicate; however, some problems arise, such as Internet addiction and even Internet violence, both of which can negatively impact students’ psychology (21, 37). In conclusion, a poor lifestyle can lead to psychological stress in students. We encourage students to develop healthy living habits as a preventive measure against psychological problems. In addition, we call on the whole society to give priority to the mental health of young people and create a favorable environment for their growth. Table 2 shows the summary information of the selected articles.

**Table 2:** The summary information of the selected articles.

Author and year	Sample characteristics	Study design	Prevalence	Assessment tools and stress level	Risk factors	Outcomes	Limitation
Zhou et al. (20)	Sample: 1,860 Grade: 7–12 Site: Shanghai China	Cross-sectional study	12.9–23.2%	Strengths and Difficulties Questionnaire (SDQ) (25.5 ± 15.5) Severe	Academic stress Criticism from others Family conflict and peer bullying and discrimination or interpersonal conflict	Negative life events are one of many factors associated with perceived stress and level of pro-social behavior in secondary school students	Sample cannot represent other area Cannot explain causation
Yang et al. (21)	Sample: 1,258 Age: 14–20 Site: Southern China	Cross-sectional study	Not given	DASS-21 (5.21 ± 4.11) Moderate	Mobile phone addiction	Mobile phone addiction has been identified as a salient risk factor for adolescents’ psychological stress	Cannot explain causation The generalizability of these findings is limited
Jinchang and Wei (17)	Sample: 350 Grade: 7–9 Site: Henan, China	Cross-sectional study	Not given	Mental stress scale (2.57 ± 0.68) Moderate	Campus safety atmosphere	The improvement of the campus safety atmosphere level can effectively mitigate the mental health	Cannot explain causation
Wen et al. (23)	Sample: 900 Grade: 9–12 Age:14.14 ± 1.32 Site: Jiang xi China	Cross-sectional study	24.78%	Mental Health Test (MHT) Low-severe	Academic pressure School mental health work	Students with extreme academic pressure are more likely to develop psychological stress	Cannot explain causation Anxiety and school-related factors were assessed using self-report surveys
Quach et al. (18)	Sample: 997 Grade: 10–12 Age: 16–19 Site: Beijing China	Cross-sectional study	Not given	Revised Children’s Manifest Anxiety Scale (RCMAS) Boys anxiety (38.22 ± 5.99) Girls anxiety (38.76 ± 5.85) Severe	Parent-child conflict	Greater parental warmth generally reduced adolescents’ psychopathology symptoms	Cannot explain causation Reliance on adolescents’ reports for information about both their parents’ behavior and their own symptoms
Lu and Liu (35)	Sample: 14,356 Age: 13–18 Site: China	Cross-sectional study	The psychological symptom detection rate of Chinese middle school students was 21.37%	Multidimensional Sub-health Questionnaire of Adolescents (MSQA) Not given	Unhealthy lifestyle	There was a positive correlation between unhealthy lifestyle and the occurrence of psychological symptoms, and boys are more easily influenced by lifestyles than girls	Cannot explain causation The questionnaire to estimate psychological symptoms, which are susceptible to the level of recall
Lian et al. (42)	Sample: 754 Age: 11–15 Grade: 7–9 Site: Shang Qiu and Wuhan, China	Cross-sectional study	Not given	DASS-21 (0.759 ± 0.497) Low	Mobile phone addiction	Mobile phone addiction was significantly and positively associated with psychological stress	Cannot be generalized to adolescents in other cultural backgrounds Cannot explain causation
Li et al. (40)	Sample: 1,440 Grade: 4–9 Site: Xiu Shui, China	Cross-sectional study	Not given	Social Anxiety Scale for Children (SASC) (2.4084 ± 0.7065) Low	Poverty	Poverty had a significantly direct effect on children’s anxiety and depression	Cannot explain causation Students’ mental health conditions may be underreported or overreported
Li et al. (44)	Sample: 19,487 Grade: 7–9 Site: China	Cross-sectional study	Not given	Not given	Martial conflicts Parent-child relationship Family income	Parental marital and parent-child relationships positively affected children’s mental health	Cannot explain causation Some limitations in the variable measurement
Guo et al. (41)	Sample: 1,228 Grade: 7–12 Age: 11–20 Site: Tong Ling and Wuhu from Anhui Province in China	Cross-sectional study	Not given	DASS-21 Anxiety (0.93 ± 0.60) Stress (1.11 ± 0.61) Low	Teacher-student relationship	Teacher support, as a kind of social support, can keep adolescents healthy by reducing the influence of negative emotions on the body and mind	Cannot explain causation Results cannot be generalized to students in other grades or adolescents from other cultural backgrounds
Geng and He (43)	Sample: 24,66 Age: 10–15 Site: China	Cross-sectional study	Not given	Kessler 6 rating scale (1.516 ± 0.584) Low	Academic pressure Parent-child conflict	Academic pressure and parent-child conflict was detrimental to the psychological health of all children	Could not examine how children’s relatedness with peers influenced their psychological well-being Could not control for the influence of regional heterogeneity Cannot explain causation
Zhang et al. (39)	Sample: 2,139 Age:14.67 ± 1.53 Grade: 10–12 Site: Southeast China	Cross-sectional study	Not given	DASS21 (13.80 ± 13.42) Moderate	Childhood trauma Social support Family functioning	Childhood trauma was positively associated with general distress among Chinese adolescents	Cannot explain causation Subjective self-report and recall bias should be interpreted cautiously
Li et al. (34)	Sample: 6,173 Age: 6–17 Site: Liaoning Province, located in northeast China	Cross-sectional study	26.6%	Strengths and Difficulties Questionnaire (SDQ) Moderate	Mother’s and father’s poor mental health Teacher’s poor mental health	All three caretakers have a significant negative influence on schoolchildren’s emotional well-being, in the order of mother > father > teacher	Cannot explain causation Not all teachers were measured
Chen et al. (38)	Sample: 938 Age: 13–18 Site: Guangzhou in southern China	Cross-sectional study	Not given	DASS-21 (14.82 ± 10.14) Moderate	Parents-child relationship Peer relationship	The results suggest that attachment, especially parent attachment, is helpful in enhancing students’ dispositional mindfulness, which in turn reduces psychological distress in secondary school students	Cannot explain causation This study only used self-report data from a single source
Chen (37)	Sample: 1,932 Grade: 7–9 Site: Taiwan and Tianjin in Mainland China	Cross-sectional study	Not given	BSRS-5 (33.45 ± 14.82) Severe	Cyber victimization	Cyber victimization, and parental support are important factors contributing to psychological distress among Taiwanese and Mainland Chinese adolescents	Cannot explain causation Sample may not be representative of the PRC Data were derived from students’ self-reports
Fu et al. (36)	Site: mainland China	Report	12.9–26.6%	Not given	Not given	Not given	Not given

## 4 Discussion

The main purpose of this review was to map the existing evidence on the prevalence and levels of psychological stress among adolescents in China, and to identify the risk factors associated with psychological stress among adolescents in China. From this review, we found that the prevalence of psychological stress among Chinese middle schools was between 12.9 and 26.6%. This figure is slightly higher than the prevalence of psychological stress among primary school students (10%) and similar to Chinese college students (20%) (45). A systematic review and meta-analysis of worldwide psychological stress among adolescents aged 10–19 years found a prevalence of approximately 25% in this age group (46), which is generally consistent with our study. Studies from other countries reported a prevalence of psychological stress among middle school students at 10.7% in 2007, and 7.6% in 2013 for Japan (47). These figures are lower than that of Chinese adolescents. According to the 2019 Korea Youth Risk Behavior Web-based Survey (KYRBWS), Korean adolescents showed a perceived stress rate of 39.9%, a prevalence of depressive mood of 28.2%, and a suicide attempt rate of 3.0% (48). This figure far exceeds that of Chinese teenagers. The levels of psychological stress ranged from low to severe, and we did not have a specific number of people at each level due to limitations in the number of reports. The overall results showed that the factors that contributed to psychological stress among Chinese middle school students were mainly concentrated in three aspects, school, family, and lifestyle. The same findings were found among Chinese primary school students, except that the elementary school population was more worried about exams, school bullying, and corporal punishment from teachers or parents (49). Our findings provide some suggestions for policy makers to reduce the psychological stress of Chinese secondary school students. First, the government should promote the reform of the education system, reduce the tendency to overemphasize examination results, and focus on the all-round development of students. Secondly, it should increase the investment in school mental health education, promote the popularization of mental health education courses, encourage schools to establish psychological counseling centers to provide students with professional psychological counseling services to help them solve their psychological problems and improve their emotional management and coping skills, and lastly, schools should carry out diversified extracurricular activities to enrich the after-school life of students and relieve their learning pressure.

Several studies of psychological stress in college student populations have shown that financial loans contribute to a certain extent to the psychological stress of college students. Some college students have to take out loans to pay for tuition and living expenses to complete their studies while others take out loans to consume (50, 51). Compared to college students, Chinese middle schools are compulsory to attend, and the government supports the tuition fees. Thus, most of the psychological pressure caused by the economy comes from the family finances. According to social causation theory, lower socioeconomic status triggers negative mental health outcomes (52). The family stress model of economic hardship can explain this phenomenon: it is suggested that financial strain within a family adversely affects the psychological well-being of children. This is linked to increased emotional distress among parents, a decline in warmth during parent-child interactions, and a rise in conflict between parents and children (53), thus increasing the psychological pressure on the students. Some empirical studies have shown elevated rates of mental disorders and emotional problems in individuals who undergo childhood trauma (54). Nearly 30% of mental disorders or emotional problems can be attributed to childhood trauma (55). Due to the immaturity of the mind during childhood, the negative events that occur during this period often make it difficult for students in childhood to resolve issues, and without parental and peer guidance, such effects may continue into adulthood and old age. As the primary place for students to grow and learn, the family plays an essential role for students. Parents, as the primary guardians of students, should take the responsibility for creating a healthy environment for students to grow up (56). Our findings likewise offer some suggestions for parents. First, parents should establish an open, honest and respectful communication relationship with their children and encourage them to share their confusion and stress. Through listening and understanding, parents can give students emotional support and comfort. Secondly, parents should try to create a positive and warm family environment to minimize arguments and conflicts so that their children can feel the warmth and support of home. Finally, parents should actively participate in the school’s educational activities and maintain close communication and cooperation with teachers. Through communication with teachers, parents can better understand their children’s performance and needs at school, and promptly identify and solve any psychological problems their children may face.

Compared to middle school students, the causes of psychological stress vary among college students around the world, and some studies have shown that alcohol consumption, tobacco use, and planning for the future have impacted college students’ psychological stress and anxiety (57, 58). In China, government regulations prohibit the sale of alcohol and tobacco to youth under the age of 18. Hence, Chinese youth are less likely to suffer from psychological stress caused by alcohol and tobacco use. Some psychological studies of adults have found that the main stressors for adults stem from work, income, and lifestyle habits, which are relatively different from those of middle school students (59, 60). Throughout our study, some commonalities can be found. Psychological stress is prevalent in all age groups, with stress levels ranging from low to severe. Lifestyle habits were mentioned to be related to psychological stress in all age groups, especially sedentary without exercising. Therefore, we would be interested in studying exercise as an entry point for more in-depth research.

We acknowledge assessing the quality of individual article is not a compulsory step in a scoping review. However, to minimize the bias in this review, we have defined the research question, this helps in focusing the scope of the review and minimizing bias associated with ambiguous or overly broad research objectives. Our team also developed a comprehensive search strategy, using multiple databases to ensure access to a wide range of evidence. Finally, rigorous inclusion and exclusion criteria were established, and data were extracted by three independent reviewers to minimize personal bias.

## 5 Conclusion

Through an exhaustive search of the available evidence, we found that psychological stress was prevalent in the Chinese adolescent population through survey, with the levels of psychological stress ranging from low to severe. There were three main sources of psychological stress (stressors) for students: school, family, and lifestyle. The main school-wide influencing factors were academic stress, teacher-student relationship, peer relationship, campus safety atmosphere, teachers’ mental health, and psychological education situation in schools. Family factors mainly include the parent-child relationship, the parent’s marital status, the family’s financial situation, parental mental health, and the student’s childhood experience. An unhealthy lifestyle was another factor that affected students’ psychological pressure, such as mobile phone consumption and internet addiction, sedentary and non-exercise habits, and poor eating habits. Our findings suggest the following recommendations for policy makers, schools and parents to reduce the tendency to overemphasize examination results, pay attention to the overall development of students, increase the investment in mental health education in schools, and carry out a variety of extracurricular activities to relieve students’ learning pressure. Parents should try to create a positive and warm family environment, minimize arguments and conflicts, and maintain close communication and cooperation with teachers.

## Data availability statement

The original contributions presented in the study are included in the article/supplementary material, further inquiries can be directed to the corresponding author.

## Author contributions

LH: Investigation, Software, Writing – original draft, Writing – review & editing, Data curation, Formal analysis, Project administration. AM: Conceptualization, Supervision, Writing – review & editing. MA: Conceptualization, Supervision, Writing – review & editing, Formal analysis, Methodology, Project administration, Software, Validation, Visualization. XM: Data curation, Writing – review & editing. HZ: Data curation, Writing – review & editing.
